# An overlooked hotspot: persistence of large polyspecific assemblages of threatened primates in the unprotected Yabassi Key Biodiversity Area

**DOI:** 10.1007/s10329-025-01212-5

**Published:** 2025-09-02

**Authors:** Vianny Rodel Vouffo Nguimdo, Ekwoge Enang Abwe, Nelson Ekole Betobe, Bethan Jane Morgan, Matthias Waltert

**Affiliations:** 1 Department of Conservation Biology, University of Göttingen, Göttingen, Germany; 2 Cameroon Biodiversity Association, Douala, Cameroon; 3 San Diego Zoo Wildlife Alliance, Escondido, CA USA; 4 Faculty of Natural Sciences, University of Stirling, Stirling, UK

**Keywords:** Primate community, Polyspecific associations, Yabassi Key Biodiversity Area, Human-dominated landscape, Afrotropical rainforest

## Abstract

The Gulf of Guinea rainforests, renowned for exceptional primate endemism, are increasingly fragmented and face severe hunting-induced defaunation. However, the long-term persistence of primate assemblages in these hunted forests remains understudied. The unprotected Ebo–Ndokbou–Makombe landscape, designated the Yabassi Key Biodiversity Area (YKBA), represents one of the region’s largest remaining forest tracts, yet its primate community is largely unexplored. Research and conservation efforts within this landscape predominantly focus on the Ebo block to protect its cryptic gorilla population, while Ndokbou and Makombe receive less attention. Here, we assess the persistence and polyspecific assemblages of diurnal primates across the three forest blocks. From January to December 2019, we surveyed over 1,500 km of reconnaissance walks and sighted seven IUCN Threatened and one Near-Threatened species, including chimpanzees *Pan troglodytes ellioti* (56 groups and 296 nest groups), putty-nosed monkeys *Cercopithecus nictitans* (435 groups), crowned monkeys *C. pogonias* (251), red-eared monkeys *C. erythrotis* (122), mona monkeys *C. mona* (108), Preuss’s monkeys *Allochrocebus preussi* (97), red-capped mangabeys *Cercocebus torquatus* (40), and drills *Mandrillus leucophaeus* (20). Notably, over 40% of primate encounters were polyspecific groups with up to five species. Despite conservation focus on Ebo, encounter rates, association patterns, and group sizes were comparable across Ebo, Ndokbou, and Makombe. Interestingly, primate encounter rates and assemblages in this unprotected landscape were also similar to observations in Korup National Park located within the same ecoregion. These findings underscore the critical conservation value of the YKBA and call for urgent landscape-scale interventions to mitigate hunting pressure and maintain habitat integrity.

**Electronic supplementary material:**

The online version of this article (doi:10.1007/s10329-025-01212-5) contains supplementary material, which is available to authorized users.

## Introduction

Despite their invaluable ecological and socioeconomic importance, primates all over the world are facing a severe extinction crisis mainly driven by habitat loss and degradation, and bushmeat hunting (Estrada and Garber 2022; Garber 2019). The populations of at least 75% of all primate species are now considered to be decreasing (Estrada et al. 2017). Consequently, scientific studies on primates have increased considerably over the past decades, aiming to understand the drivers of this crisis and propose science-based interventions to reverse the declining trend of primate populations (Bezanson and McNamara 2019). However, there has also been considerable bias regarding the conservation attention given to some species and regions (Junker et al. 2020). For instance, great apes have benefited from a disproportionately high research attention compared to other species, with no correlation to their extinction risk (Bezanson and McNamara 2019; Marshall and Wich 2016). Most research has also taken place in protected areas, which are considered the cornerstone of biodiversity conservation (Bezanson and McNamara 2019) despite the fact that unprotected areas are increasingly recognized as critical for primate conservation (Estrada et al. 2012). With the ongoing habitat fragmentation and incessant land use pressure for economic purposes, the success of primate conservation will likely rely on complex multi-use landscapes (Marshall and Wich 2016). Yet, there is still a lack of basic information on the status of the primate community in most of these human-dominated landscapes (Chazdon et al. 2009; Galan-Acedo et al. 2019), some of which are globally recognized as critical areas for biodiversity.

The Gulf of Guinea is a global biodiversity hotspot characterized by high levels of endemism, including many primate species (Linder and Oates 2011; Oates et al. 2004). Despite this biological importance, the area is increasingly fragmented with only a few remaining large forest blocks (Fonteyn et al. 2023). The Ebo–Ndokbou–Makombe landscape, designated as the Yabassi Key Biodiversity Area (YKBA) (Key Biodiversity Areas Partnership 2025), is one of those last remaining forest tracts in this ecoregion (Bowers-Sword 2020; Potapov et al. 2017). Along with its Key Biodiversity Area status, the YKBA is also recognized as an Important Bird Area (Whytock et al. 2014), and part of it is listed as a Tropical Important Plant Area (Murphy et al. 2021). Furthermore, it is entirely or partially documented as one of the priority areas for the conservation of several highly threatened primate species, including the Nigeria-Cameroon chimpanzee *Pan troglodytes ellioti*, the red-capped mangabey *Cercocebus torquatus*, and the drill *Mandrillus leucophaeus* (Dempsey et al. 2024; Linder et al. 2021b; Morgan et al. 2011, 2013). But despite this biodiversity importance, the YKBA is not protected and is largely dominated by human activities, including industrial and artisanal logging and agriculture (Mahmoud et al. 2019), and rampant hunting and bushmeat trade, putting some of its most iconic primate species, such as the Preuss’s red colobus (*Piliocolobus preussi*) to the brink of local extinction (Linder et al. 2021a; Nguimdo et al. 2025). Meanwhile, the YKBA as a whole has been quite understudied, and its primate community is still poorly known. The Ebo forest block has received more research attention, but this has focused either on great apes (Abwe et al. 2020, 2019; Mfossa et al. 2019) or on the large mammal community (Whytock et al. 2021), with field methods failing to appropriately detect, document, and describe the entire arboreal and terrestrial primate community. The only recent survey in the Ndokbou forest block was carried out by Bowers-Sword (2020) along a total of ca. 200 km of reconnaissance walks (hereafter referred to as recces). Consequently, most conservation attention has also focused solely on Ebo (Abwe et al. 2015; Mfossa et al. 2025). Basic scientific knowledge of the primate community of the YKBA is urgently needed to inform conservation strategies that engage conservation actors and local communities to conserve the biodiversity and the ecological integrity of the landscape as a whole. This paper aims to contribute to filling this critical knowledge gap.

From January to December 2019, we surveyed the Ebo, Ndokbou, and Makombe forest blocks (hereafter referred to as Ebo, Ndokbou and Makombe, respectively) along more than 1500 km of recces, focusing primarily on their primate communities. Here, we used the data collected during those surveys to (i) assess and compare the diversity and relative abundance of diurnal primate species across the three forest blocks which compose the YKBA, (ii) explore the polyspecific assemblages of primate species in the landscape, and (iii) examine how these results compare to findings from protected and unprotected areas within the Gulf of Guinea ecoregion. We expected primate abundance to be higher in Ebo compared to Ndokbou and Makombe, as Ebo has been the location of almost all conservation attention in the landscape for over two decades (Abwe and Morgan 2008; Mfossa et al. 2025). We also expected primate diversity and encounter rates to be lower in this human-dominated landscape compared to protected areas in the same ecoregion (Linder and Oates 2011).

## Methods

### Study area

**Fig. 1:**
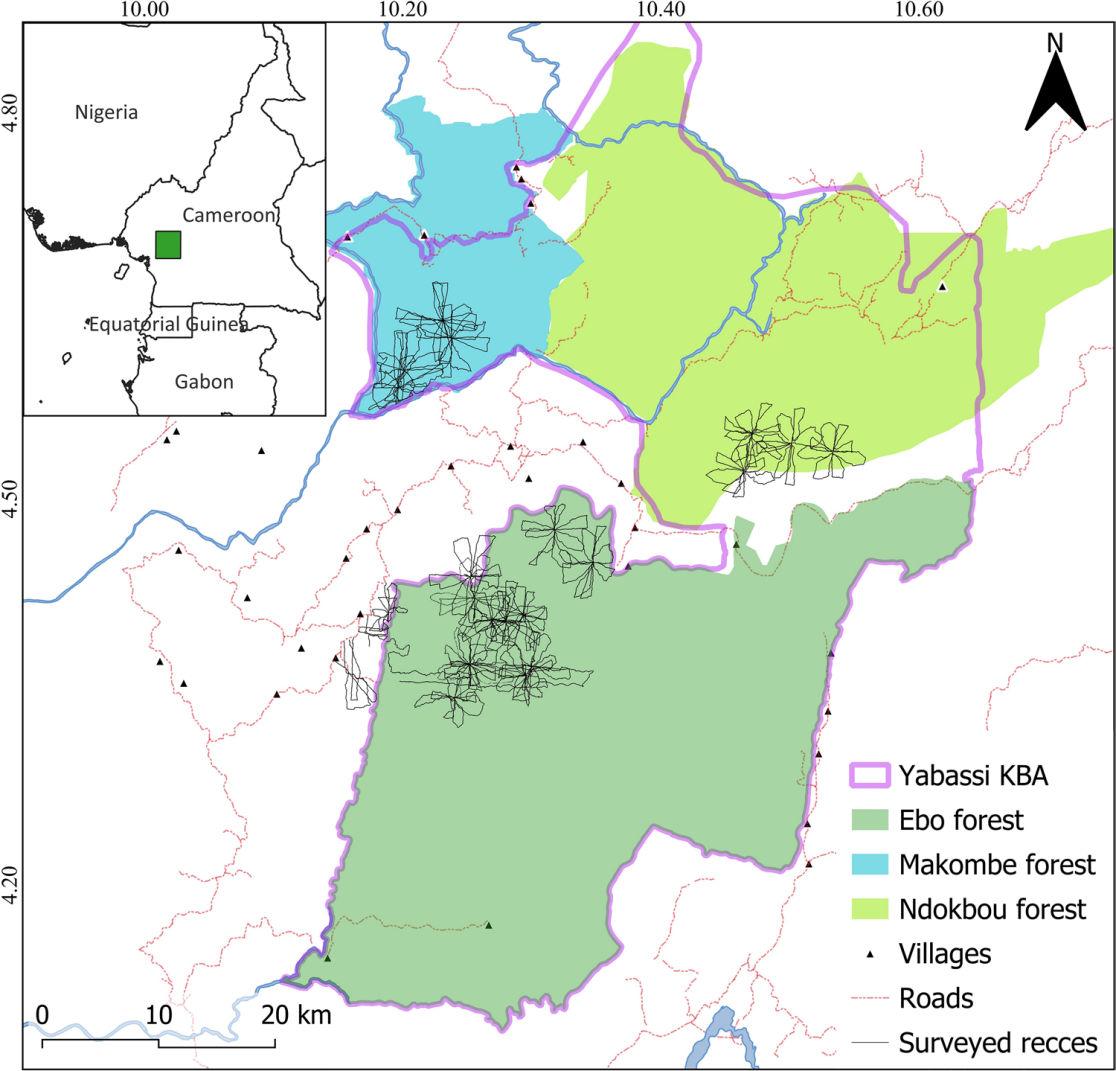
Map showing the location of Yabassi Key Biodiversity Area in Central Africa, as well as the Ebo, Ndokbou, and Makombe forest blocks with the recces (GPS tracklogs) surveyed from January to December 2019

The YKBA is located in southwestern Cameroon, north of the Sanaga River, the biogeographical divide between the Congo Basin and the Gulf of Guinea rainforests (Fig. 1). It spans more than 3000 km^2^ and is composed of three forest blocks, namely Ebo (1400 km^2^), Ndokbou (1000 km^2^) and Makombe (600 km^2^). The area is characterized by open and closed canopy lowland and sub-montane rainforest, ranging in altitude from as low as 30 m to over 1300 m (Abwe 2018; Bowers-Sword 2020). Elevations in Ebo (mean = 510 m, range 30–1280 m) and Ndokbou (mean = 406 m, range 85–1329 m) are considerably higher than that of Makombe (mean = 264 m, range 64–622 m). The climate of the landscape is equatorial with two main seasons: the rainy season from March to November and the dry season from December to February (Abwe 2018). The total annual rainfall in the area is estimated to range between 2000 and 3000 mm (Abwe et al. 2019; Mahmoud et al. 2019). Due to its proximity to the major urban centers in Cameroon including Douala, Edea, and Yaoundé, the YKBA is under very intense pressure from hunting and logging activities (Bowers-Sword 2020; Mfossa et al. 2022; Nguimdo et al. 2025). While industrial logging has been ongoing in Ndokbou and Makombe for many years, Ebo was partially and selectively logged in the 1970s. Consequently, the three forest blocks are made of a mix of young and mature secondary forests in areas that were logged in the past, and the most rugged areas that were not affected by logging still harbor mature vegetation (Abwe 2010; Bowers-Sword 2020). Ebo was proposed as a protected area in 2006, but in 2020, the Government of Cameroon changed its plans and decided to turn it into two concessions (Nanda et al. 2023; Whytock et al. 2021). Despite global opposition to the logging project, it was classified into two forest management units in 2023 (Nguimdo et al. 2025), after the data presented here were collected. Nevertheless, YKBA is recognized for its outstanding biodiversity which includes a wide range of plants, many of which are endemic or nearly endemic to the landscape (Cheek et al. 2022, 2018), and several wildlife species, including one of the main remaining populations of the Nigeria-Cameroon chimpanzees (*Pan troglodytes ellioti*), a small and geographically isolated population of gorillas (*Gorilla gorilla*) in Ebo, the last remaining population of African forest elephants (*Loxodonta cyclotis*) in the Littoral Region of Cameroon, and an understudied community of arboreal and semi-terrestrial non-hominid primates (Abwe 2018; Mfossa et al. 2022; Whytock et al. 2021).

### Primate data collection

We carried out monthly field surveys from January to December 2019 in Ebo, Ndokbou, and Makombe (Fig. 1). Each monthly trip lasted on average 12 days and included two survey teams, each comprising two well-trained data collectors with experience in primate identification and survey techniques. During each trip, the entire survey crew traveled to one central camping point, usually near a water source, from where the teams started the survey during the subsequent 4 days. Every morning, the surveys started around 07:00, and the two teams left the camp following opposing cardinal directions, walking at a speed of approximately 1 km/h to avoid disturbing animals as much as possible. Daily survey tracks followed a triangular pattern. For instance, on the first day, Team A might head north until 12:00, then deviate east until 14:00, and finally return to camp. On another day, when Team B begins by heading north, they would slightly adjust their trajectory to avoid overlapping with the previous track of Team A. At 12:00, they would instead turn west, continue until 14:00, and then return to camp. After 4 days at the first camping site, each team had surveyed each of the four cardinal directions once (Supplementary Material). The survey crew then moved to a new camping site away from the first and repeated the surveys for four additional days before leaving the forest.

For each primate group encountered, we stopped the survey to observe and describe them carefully and quietly. We recorded the geographic coordinates of the observation, the type of the sign (direct sightings or vocalizations), the group composition (primate species, number of individuals per species), their activity (feeding, resting, travelling), and the approximate height range of the species in the canopy (Gonzalez Kirchner 2004; Sheth 2006). When chimpanzee nests were encountered, we also stopped the surveys to search for additional nests of the same age within 30 m of the initial nest (Abwe 2018; Koops et al. 2012), and recorded the GPS coordinates of the center of the nest group and other information including the estimated age of the nests based on the inspection of the nest construction material. When a group had nests of different ages, we considered all the nests with the same age as a different group (Koops et al. 2012).

### Data preparation and analysis

As it is often difficult to count all the individuals in primate groups in tropical rainforests reliably, we focused our analyses on primate groups as opposed to individuals. Nevertheless, for sighted primate groups, we used the number of observed individuals to assess the average group size of each species. We used both direct sightings and vocalizations to determine the number of encounter events with each primate species, but only used direct sightings to analyze sighting frequency and polyspecific associations patterns. We overlaid a system of 1 × 1 km grid cells across the entire area, counted the number of encounter events in each surveyed grid cell, and divided it by the distance walked within that grid cell to obtain the encounter rate of each species. For chimpanzees, we included nest groups in the computation of their encounter rate. We then grouped the grid cells and the corresponding encounter rates of different species based on their respective forest block (Ebo, Ndokbou or Makombe), and we used the Kruskal–Wallis test to compare the encounter rate of primate species between the three forest blocks. We further used the pairwise Wilcoxon rank-sum test to check statistical differences between those areas.

To examine multispecies grouping patterns within the primate community of YKBA, we first created a species presence-absence matrix, considering both monospecific and polyspecific primate groups (only direct sightings) as clusters (rows), with the different species in the columns. For example, the value ‘1’ in cluster ‘i’ of column ‘j’ indicates that species ‘j’ was present in cluster ‘i’, while the value ‘0’ denotes its absence (Astaras et al. 2011). Using the resulting matrix, we determined the frequency of encounter events with each primate species when recorded alone or when associated with one or more additional species. To investigate the strength of associations between different pairs of species, we computed the Dice’s coefficient, which measures the degree of coexistence between two species A and B over different localities (Janson and Vegelius 1981). It uses the formula:

$$\text{Dice}=\frac{2a}{\left(2a+b+c\right)}$$

where: a: the number of localities occupied both by A and B; b: the number of localities that only have A; c: the number of localities that only have B (Janson and Vegelius 1981; Zhang and Ma 2013).

In our case, we considered our clusters (primate groups) as the different localities (Astaras et al. 2011). In addition to the Dice’s index, we also computed other parameters indicating the degree of association between each pair of species, including the Jaccard index and the Ochiai index (Supplementary Material), using the species association analysis package ‘spaa’ version 0.2.5 (Zhang and Ma 2013).

To classify primate species based on their recorded position along the vertical gradient of the forest canopy, we used the following categorization: species observed with most individuals on the forest floor or below 5 m were designated as occupying the "lower canopy"; those found between five and 15 m were classified as occupying the "mid-canopy"; and species recorded above 15 m were described as occupying the "upper canopy". We then analyzed and compared the species by calculating the percentage of encounter events of each species within each of the three defined canopy ranges. All statistical analyses were implemented using R 4.5 (R Core Team 2025).

## Results

### Primate community

**Fig. 2:**
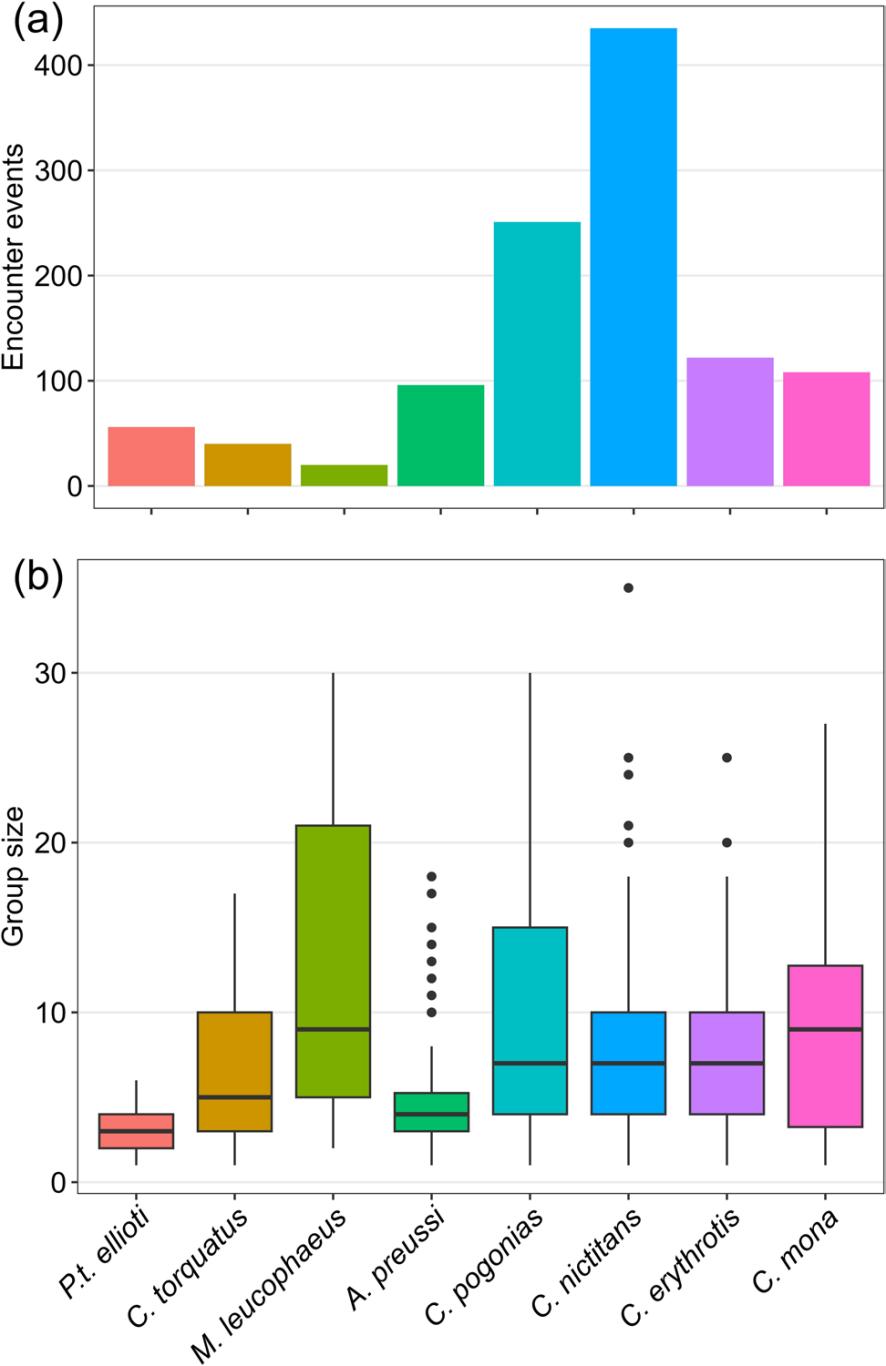
Encounter events (including sightings and calls) and observed group size (sightings only) of the primate species recorded during surveys

Our total survey effort was 1509 km, with 950 km in Ebo, 257 km in Ndokbou, and 302 km in Makombe. We documented a total of 359 signs of the Nigeria-Cameroon chimpanzees (*Pan troglodytes ellioti*), including nine direct sightings, 47 vocalizations, and 296 nest groups of varying ages, with nest group sizes ranging from one to 21 individual nests. Additionally, we recorded seven non-hominid diurnal primate species (Fig. 2a, Table 1), including putty-nosed monkeys (*Cercopithecus nictitans ludio*) with 435 groups (0.29 groups/km), crowned monkeys (*Cercopithecus pogonias pogonias*) with 251 groups (0.17 groups/km), red-eared monkeys (*Cercopithecus erythrotis camerunensis*) with 122 groups (0.08 groups/km), mona monkeys (*Cercopithecus mona*) with 108 groups (0.07 groups/km), Preuss’s monkeys (*Allochrocebus preussi preussi*) with 96 groups (0.06 groups/km), red-capped mangabeys (*Cercocebus torquatus*) with 40 groups (0.03 groups/km), and drills (*Mandrillus leucophaeus leucophaeus*) with 20 groups (0.01 groups/km). There was considerable variability in group sizes across these species, with the largest mean group sizes observed for the mona monkey and the drill, and the smallest for chimpanzees and Preuss’s monkey (Fig. 2b).

**Table 1:** Mean encounter rates (groups/km) and standard error (SE) of primate species recorded during the survey in the Yabassi Key Biodiversity Area, with Kruskal–Wallis test results on the difference between forest blocks

Primate species or subspecies recorded in YKBA	IUCN status	Mean encounter rate (groups/km) ± SE		
		Ebo	Ndokbou	Makombe
*Pan troglodytes ellioti*	EN	0.31 ± 0.04^a^	0.25 ± 0.05^a^	0.04 ± 0.01^b^
*Cercocebus torquatus*	EN	0.04 ± 0.01^a^	0.05 ± 0.04^a^	0.00 ± 0.00^b^
*Mandrillus leucophaeus leucophaeus*	EN	0.01 ± 0.01^a^	0.01 ± 0.01^a^	0.03 ± 0.01^b^
*Allochrocebus preussi preussi*	EN	0.06 ± 0.01^a^	0.08 ± 0.02^a^	Not detected
*Cercopithecus pogonias pogonias*	VU	0.17 ± 0.02	0.19 ± 0.05	0.10 ± 0.02
*Cercopithecus nictitans ludio*	VU	0.30 ± 0.03^a^	0.37 ± 0.06^a^	0.16 ± 0.03^b^
*Cercopithecus erythrotis camerunensis*	VU	0.08 ± 0.01^a*^	0.12 ± 0.03^a^	0.04 ± 0.01^b*^
*Cercopithecus mona*	NT	0.04 ± 0.01^a^	0.07 ± 0.02^ab^	0.11 ± 0.03^b^

The significance of the difference between sites is derived from the pairwise Wilcoxon rank-sum test and indicated by superscript notation

IUCN Red List status: *VU* vulnerable, *EN* endangered, *NT* near threatened

Superscript letters (a and b) indicate results from pairwise Wilcoxon rank-sum tests; values sharing the same letter are not significantly different (*p* > 0.05), while values with different letters are significantly different (*p* < 0.05)

*Marginal significance *p* = 0.057

### Primate occurrence in different blocks of the YKBA

**Fig. 3:**
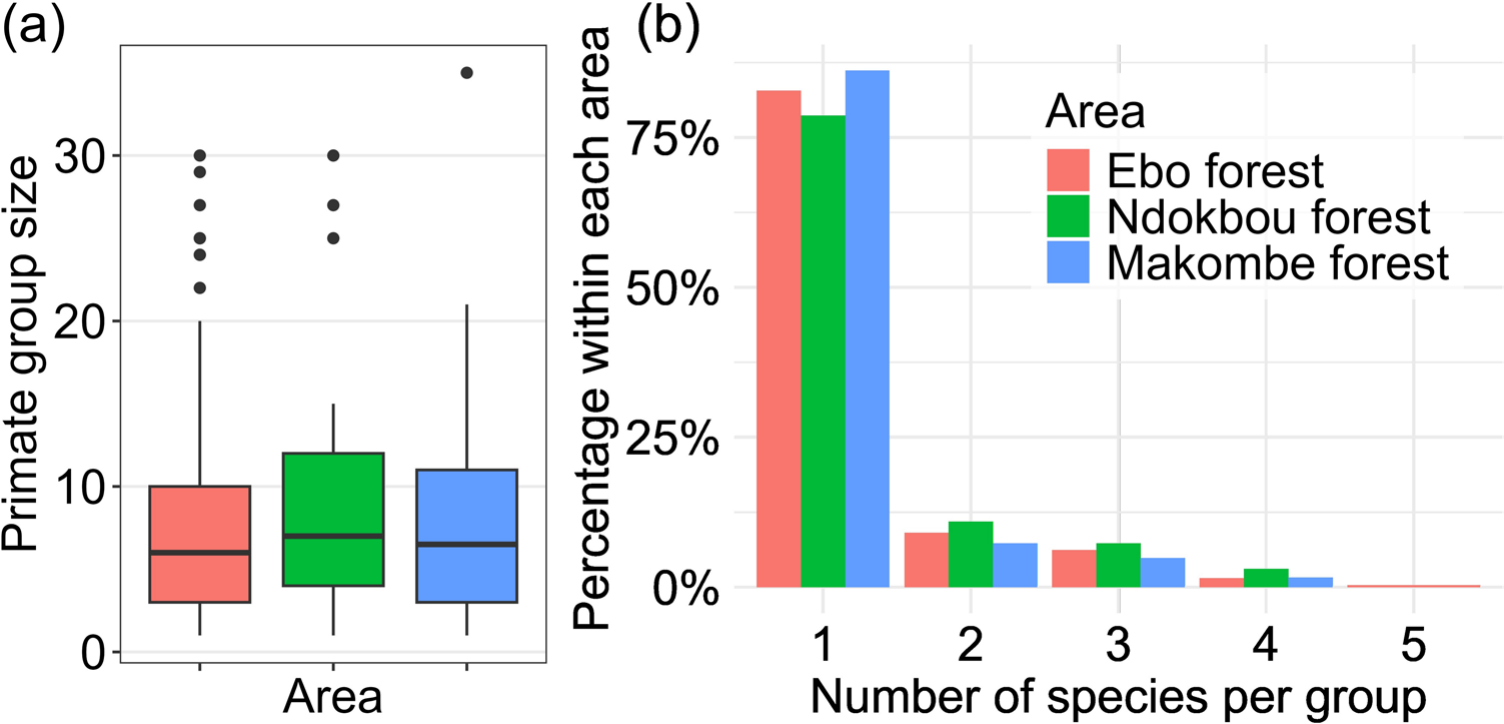
Proportion of mono and polyspecific primate encounters recorded in the three forest blocks of Yabassi Key Biodiversity Area during the surveys

With the exception of Preuss’s monkey *A. preussi*, which was not recorded in Makombe, all other seven primate species were observed in Ebo, Ndokbou, and Makombe (Table 1). However, encounter rates for these species varied substantially between the three forest blocks. Chimpanzee *P.t. ellioti* encounter rates were significantly (nearly ten times) lower in Makombe compared to Ebo and Ndokbou, a trend also observed for the putty-nosed monkey *C. nictitans* and the red-capped mangabey *Cercocebus torquatus*. Conversely, the drill *M. leucophaeus* exhibited a significantly higher encounter rate in Makombe compared to the other two forest blocks. Additionally, the encounter rate of the mona monkey *C. mona* was significantly higher in Makombe than in Ebo. The red-eared monkey *C. erythrotis* had significantly higher encounter rates in Ndokbou compared to Makombe, which, in turn, had marginally lower encounter rates relative to Ebo (*p* = 0.05683). Notably, the encounter rates of the crowned monkey *C. pogonias* did not vary significantly across the three forest blocks. The overall group size of encountered primate groups was relatively similar across the three forest blocks (Fig. 3a).

### Primate polyspecific associations

**Fig. 4:**
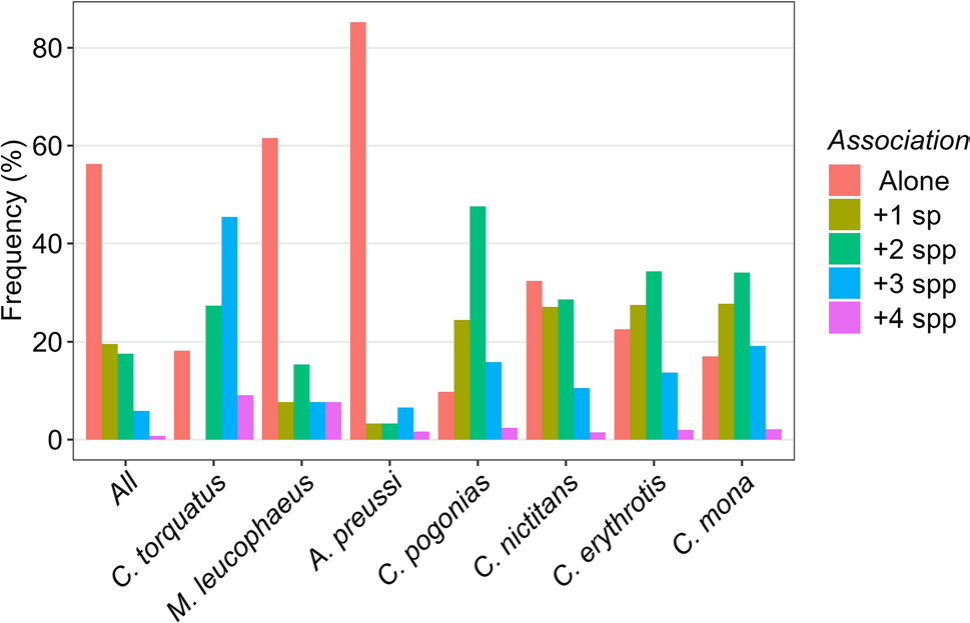
Proportion of mono and polyspecific occurrence in the different primate species recorded in the Yabassi Key Biodiversity Area during the surveys

Nearly 60% of all direct encounters with primate species involved monospecific groups (Figs. 3b, 4). Although we only observed polyspecific groups comprising up to five species in Ebo (*n* = 2), the proportion of groups containing a given number of species was relatively similar across the three forest blocks (Fig. 3b).

The high proportion of monospecific groups was primarily driven by the Preuss’s monkey *A. preussi* and the drill *M. leucophaeus*, both of which were predominantly observed alone (Fig. 4). In contrast, other primate species typically occurred in association with at least one additional species. Among the arboreal species, the putty-nosed monkey *C. nictitans* exhibited the highest relative frequency of monospecific groups. The red-capped mangabey *Cercocebus torquatus* and the crowned monkey *C. pogonias* were most frequently observed in association with two or more additional species (Fig. 4). Chimpanzees *P.t. ellioti*, however, were encountered infrequently (*n* = 9) and exclusively in monospecific groups, leading to their exclusion from the association analyses.

The surveys revealed considerable dyadic associations among the arboreal primate species (Table 2). Specifically, the putty-nosed monkey *C. nictitans* exhibited frequent associations with the red-eared monkey *C. erythrotis* (60 encounter events) and with the crowned monkey *C. pogonias* (59 encounter events), while the crowned monkey also demonstrated associations with the red-eared monkey (48 encounter events). Furthermore, all three species were observed co-occurring within polyspecific groups on 35 occasions, with the highest association coefficients (Dice > 0.5). A relatively stronger association was also found between the mona monkey *C. mona* and these three species (Dice ≥ 0.3).

**Table 2:** Number of encounter events (*n*) and Dice’s index (Dice) showing the degree of association between each pair of primate species

*n*	Dice						
	*Allochrocebus preussi*	*Cercopithecus erythrotis*	*Cercopithecus mona*	*Cercopithecus nictitans*	*Cercopithecus pogonias*	*Cercocebus torquatus*	*Mandrillus leucophaeus*
*Allochrocebus preussi*	***N* = 60**	0.07	0.00	0.07	0.10	0.03	0.03
*Cercopithecus erythrotis*	6	***N* = 102**	0.34	0.51	0.52	0.12	0.05
*Cercopithecus mona*	0	25	***N* = 47**	0.28	0.36	0.10	0.03
*Cercopithecus nictitans*	7	60	25	***N = *132**	0.55	0.10	0.07
*Cercopithecus pogonias*	7	48	23	59	***N* = 82**	0.15	0.04
*Cercocebus torquatus*	1	7	3	7	7	***N* = 11**	0.00
*Mandrillus leucophaeus*	1	3	1	5	2	0	***N* = 13**

The bold numbers (*N*) represent the total number of direct sightings of the species

### Vertical stratification of primate species

**Fig. 5:**
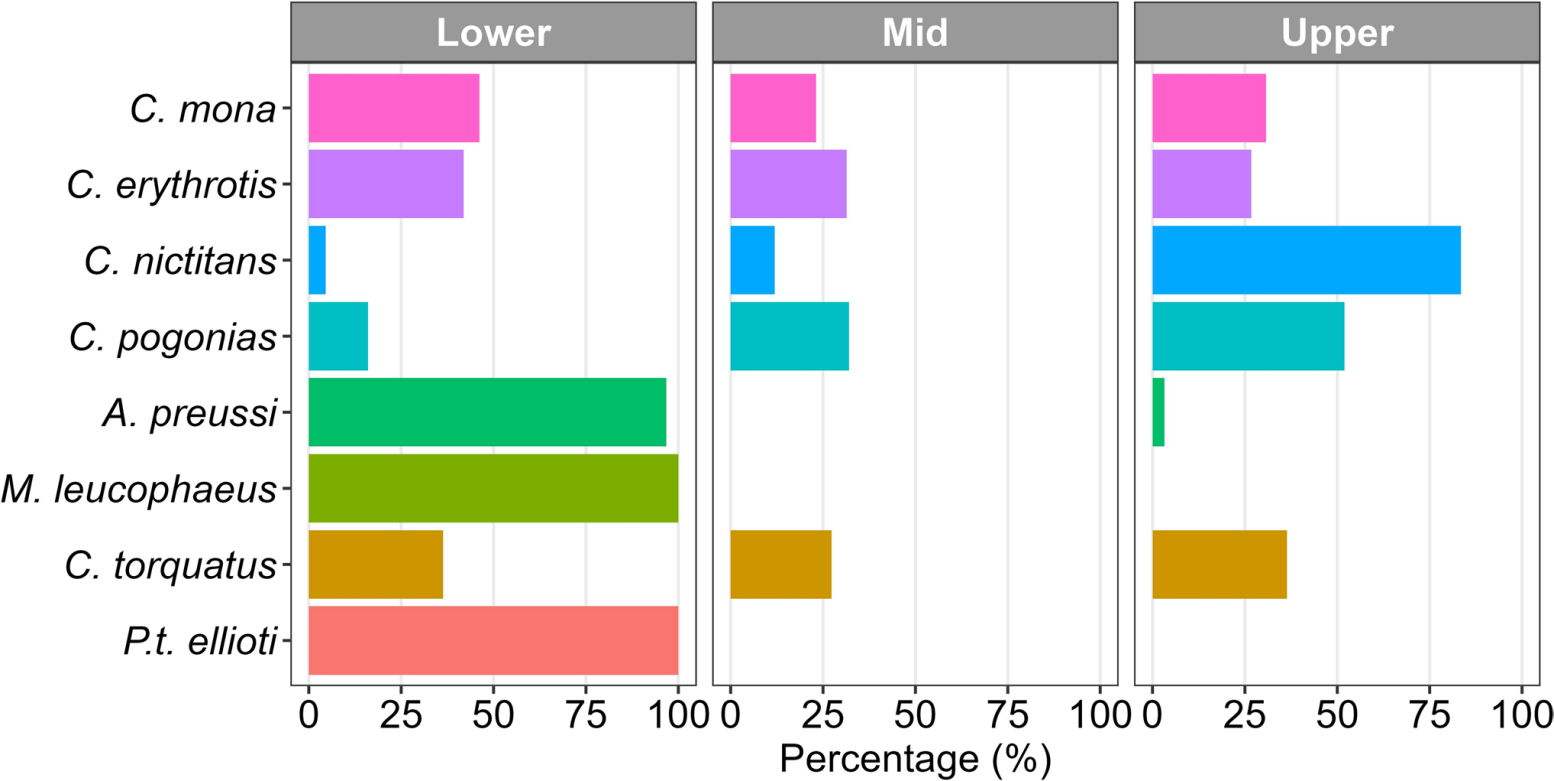
Frequency of primate sightings in the different layers of the forest canopy: Lower (0–5 m), Mid (5–15 m) and Upper (> 15 m)

Within mono and polyspecific groups, the Preuss’s monkey *A. preussi* and the drill *M. leucophaeus* predominantly occupied the lower strata of the canopy (Fig. 5). In contrast, the putty-nosed monkey *C. nictitans* primarily occurred in the upper layers of the canopy, often positioning itself above all other species within polyspecific groups. A similar pattern was observed for the crowned monkey *C. pogonias*, but the species was also often observed in the lower strata. The red-eared monkey *C. erythrotis*, the mona monkey *C. mona*, and the red-capped mangabey *Cercocebus torquatus* exhibited greater variability in their position in the canopy (Fig. 5).

## Discussion

The Gulf of Guinea is considered a hotspot of hunting-induced mammal defaunation, hosting several bushmeat markets where threatened primate species are traded (Benitez-Lopez et al. 2019; Fa et al. 2014). There is therefore an urgent need to protect the remaining populations of primates across this ecoregion, especially those residing in unprotected landscapes, which are generally more vulnerable to hunting compared to protected areas (Rovero et al. 2015). However, we still know very little about primate persistence in these human-dominated areas, and this lack of knowledge hinders conservation action. As one of the few remaining large forest tracts in the Gulf of Guinea (Potapov et al. 2017), the YKBA may play a major role in ensuring the survival of the threatened primate species of the ecoregion. This study represents the first and most comprehensive effort to assess the persistence and assemblage of diurnal primate species across the entirety of the YKBA. It complements the surveys carried out by Bowers-Sword (2020) which were limited to a relatively small area of Ndokbou. By exploring the entire landscape, we also shed light on Makombe, which has remained largely overlooked by conservation scientists and practitioners compared to Ebo.

### Recce surveys to assess primate communities

While our field survey method using recce surveys does not permit us to estimate species densities, it serves as a valuable initial exploration, offering an overview of the status of the primate community of a largely unknown forested landscape in the Gulf of Guinea ecoregion (Kühl et al. 2008; Plumptre et al. 2013). Previous studies have used this method to assess primate communities in various regions, either as the sole approach (Bowers-Sword 2020; Mfossa et al. 2022) or in combination with other techniques such as line transects (Binczik et al. 2017; Fotang et al. 2023; Strindberg et al. 2018). Recce surveys are a cost-effective means of evaluating wildlife communities over large and rugged areas where implementing line transects or other survey methods may be prohibitively expensive and are particularly valuable in regions where wildlife densities are so low that alternative methods fail to yield detections (Plumptre et al. 2013; Strindberg et al. 2018; Zausa et al. 2023). They also have the advantage of causing less damage to the vegetation as opposed to line transects, which require opening transects prior to surveys (White and Edwards 2000). The results from studies based on recce surveys have been used to inform the conservation status of threatened species such as the Critically Endangered Preuss’s red colobus (Linder et al. 2021a, 2021b).

Even when recce surveys are employed solely to estimate species encounter rates, there remains potential for bias stemming from the field survey approach. Such bias may arise from, for instance, following pre-existing human or wildlife trails, or from non-representative sampling caused by altering survey direction in response to terrain heterogeneity (Kühl et al. 2008; White and Edwards 2000). In this study, we deliberately avoided established trails and, where feasible, used pruning shears to cut through dense vegetation. By adopting survey directions based on cardinal directions, therefore minimizing deviations (Supplementary Material), our approach is similar to the ‘guided’ recces, which are considered less prone to directional bias, and therefore more representative of the surveyed area (Kühl et al. 2008). Deviations from the planned path were only made when natural obstacles, such as cliffs or ravines, made progression impossible. Our field approach may have also introduced some bias related to changes in the detection probability along the recces, as we did not conduct observations within a fixed strip width nor measure perpendicular distances from the recce line to observed primate groups (Kühl et al. 2008; Plumptre et al. 2013). To attenuate this, we employed a team of highly experienced primate field researchers who had participated in previous primate surveys within the same ecoregion. Additionally, we ensured consistency in data collection and observer-related bias by retaining the same survey teams throughout the entire survey period (Whytock et al. 2021). Finally, within each forest block, the surveyed sites were not fully representative of the entire block, as they were often clustered in one region due to logistical constraints. Some sites were also surveyed more than once, but we ensured that repeated visits did not occur in consecutive months. Despite these limitations and given our effort to mitigate them, we believe that our results offer a reliable overview of the primate community within this largely understudied landscape and can serve as a foundation for future surveys employing more standardized methodologies.

### Significance of YKBA for primates in the Gulf of Guinea

Across the YKBA, we recorded eight of the ten diurnal non-hominid primate species known to be present in the landscape; the same species reported by Bowers-Sword (2020) after surveying ca. 200 km of recces in Ndokbou. Four of the eight primate species recorded in the YKBA are listed as Endangered on the IUCN red list, three are listed as Vulnerable, and one is listed as Near-Threatened (IUCN 2025). Similarly, Astaras et al. (2011) found the same number of species in the Korup National Park, located in the same ecoregion near the Nigeria-Cameroon border, but instead of the Preuss’s monkey *Allochrocebus preussi*, which likely does not exist inside the park, they recorded the Preuss’s red colobus *Piliocolobus preussi*, which we failed to detect during our surveys. The Preuss’s red colobus has not been observed by researchers in the YKBA for more than a decade (Linder et al. 2021a). However, in Ndokbou, Bowers-Sword (2020) recorded what seemed to be the vocalization of the species, but only on one occasion during three months of surveys. Given the conservation importance and the Critically Endangered status of the species (Linder et al. 2024), we highly recommend further surveys combining local ecological knowledge and additional survey techniques such as arboreal camera trapping and intensive passive acoustic monitoring. During our surveys, we also failed to detect gorillas as our surveys did not cover the approximately 40 km^2^ area known to be inhabited by the small population of gorillas in the north of Ebo (Mfossa et al. 2022). Overall, primate species richness in YKBA was among the highest across the region, as primate surveys in other areas generally yield lower primate richness, as evidenced by the results from Angwafo et al. (2019) in Mount Kupe, Cameroon, Orimaye et al. (2022) in Akure Forest Reserve, Nigeria, and Cronin et al. (2016) and Forrest et al. (2017) on Bioko Island, Equatorial Guinea.

As reported by Bowers-Sword (2020) in Ndokbou and by other studies in the same ecoregion (Astaras et al. 2011; Linder and Oates 2011; Waltert et al. 2002), the putty-nosed monkey *C. nictitans* was the most common of all primate species encountered. The drill *M. leucophaeus* and the red-capped mangabey *Cercocebus torquatus* were the least recorded species during the surveys with only 20 and 40 groups encountered respectively, including direct sightings and vocalizations. Nevertheless, the mean encounter rates (sightings and vocalizations) of non-hominid primate species from this study were very similar to or even higher than observations from Korup National Park during surveys between 2006 and 2008 (Astaras et al. 2011). In addition, when we excluded the vocalizations from the analyses, the primate sighting frequency from this human-dominated landscape was only slightly lower for some species compared to the observations in Korup National Park during 2004–2005 by Linder and Oates (2011) (Table 3). For chimpanzees *P.t. ellioti*, the surveys carried out by Kupsch and Bobo (2024) in 2014 in the Banyang Mbo Wildlife Sanctuary reported a mean encounter rate of 0.129 per km (nests and dung) and 0.085 encounters per km in Korup National Park, with no direct sightings nor vocalizations in both sites (Table 3). Both values are considerably lower than the 0.306 encounters per km in Ebo and 0.248 encounters per km in Ndokbou, and the overall 0.238 encounters per km in the YKBA. Primate encounter rates in YKBA are also higher than results from a logging concession and an agroforestry matrix near Korup National Park (Kupsch et al. 2023). These findings suggest that despite the high and increasing hunting intensity in this unprotected and multi-use landscape (Bowers-Sword 2020; Nguimdo et al. 2025), its primate community has largely succeeded in surviving and is performing almost as well as the primate populations of formally protected areas. These results underscore the importance of the YKBA as a stronghold for primate conservation, critical for their survival in the Gulf of Guinea as a whole.

**Table 3:** Sighting frequency (number of primate groups directly sighted per km) from this study, from Linder and Oates (2011) in the south-western (SW) and north-eastern (NE) parts of Korup National Park, and from Kupsch and Bobo (2024) in the Banyang Mbo Wildlife Sanctuary (BMWS, including direct sightings and calls)

	From this study in 2019			From Linder and Oates (2011) in 2004–2025		From Kupsch and Bobo (2024)^a^	
	Ebo	Ndokbou	Makombe	Korup SW	Korup NE	BMWS	Korup
*Pan troglodytes*	0.01	0.01	0.01	Excluded	Excluded	0.00	0.00
*Cercocebus torquatus*	0.01	0.01	0.00	0.01	0.00	0.00	0.05
*Mandrillus leucophaeus*	0.01	0.01	0.01	0.01	0.00	0.00	0.00
*Allochrocebus preussi*	0.05	0.07	0.00	/	/	0.00	/
*Cercopithecus pogonias*	0.06	0.06	0.04	0.05	0.04	0.00	0.06
*Cercopithecus nictitans*	0.09	0.13	0.05	0.19	0.23	0.23	0.45
*Cercopithecus erythrotis*	0.07	0.10	0.02	0.10	0.05	0.16	0.24
*Cercopithecus mona*	0.02	0.03	0.06	0.04	0.09	0.23	0.30

^a^Kupsch and Bobo (2024) included both direct sightings and vocalisations in the analyses

### Primate encounter rates in different blocks of the YKBA

Ebo, Ndokbou, and Makombe had very similar primate encounter rates. Apart from the Preuss’s monkey *A. preussi*, which was not recorded in Makombe, all other species recorded during our surveys occurred in the three blocks. The absence of the Preuss’s monkey in Makombe may be related to the lower elevations in the surveyed part of this forest block (mean elevation, Ebo = 484 m, Ndokbou = 739 m and Makombe = 188 m) as this species is known to mainly occur at high elevations (Gonzalez Kirchner 2004). The mona monkey *C. mona*, which is suggested to be highly resilient to hunting (Linder and Oates 2011), was significantly more commonly encountered in Makombe compared to Ebo. Conversely, the encounter rate of chimpanzee *P.t. ellioti* was lowest in this forest block. Makombe has an increased proximity to roads and villages with higher accessibility to Douala, a major city in the region and a hotspot of bushmeat trade (Bowers-Sword 2020; Fa et al. 2014). The part of Makombe where the surveys took place is also characterized by less rugged terrain (mean terrain ruggedness index, Ebo = 26.12, Ndokbou = 28.60 and Makombe = 18.62) compared to the two other forest blocks, which may mean that it is more accessible to humans and therefore experiences more bushmeat hunting pressure (Nguimdo et al. 2025).

The mona monkey *C. mona* and the putty-nosed monkey *C. nictitans* are usually hypothesized to benefit from competition release in areas with high bushmeat hunting leading to the depletion of other primate species (Linder and Oates 2011; Waltert et al. 2002). This seemed to be true for the mona monkey, which was significantly more common in Makombe and appeared to have the largest mean group size of all arboreal primate species. Conversely, we found that the putty-nosed monkey was significantly less common in Makombe compared to the two other blocks. Nevertheless, it was the most common of all primate species in YKBA (Waltert et al. 2002), suggesting that it may also be relatively more resilient to hunting (Linder and Oates 2011). Our results revealed that Ebo and Ndokbou had the highest mean encounter rates for the chimpanzee *P.t. ellioti*, the putty-nosed monkey *C. nictitans*, and the red-capped mangabey *Cercocebus torquatus*, while Makombe had significantly higher mean encounter rates for the drill *M. leucophaeus* and the mona monkey *C. mona*. We found no significant difference in the encounter rates of the crowned monkey *C. pogonias* among the three forest blocks. These results show that while the current focus of conservation interventions and research has solely been on Ebo (Abwe et al. 2015; Mfossa et al. 2025; Whytock et al. 2021), Ndokbou and Makombe are also very important for primates in the landscape. But this is often overlooked in primate conservation planning in the region. For instance, the recently published Cercocebus and Mandrillus action plan lists Ebo and the Korup National Park as the only key conservation priority areas in Cameroon for drills (Dempsey et al. 2024), while our findings show significantly higher encounter rates of the species in Makombe and similar encounter rates in Ebo and Ndokbou. In addition, the Nigeria-Cameroon chimpanzee action plan also prioritizes Ebo over Ndokbou (Morgan et al. 2011), while our results show statistically similar encounter rates for the species in the two blocks. Moreover, on the IUCN red list, the distribution of the Preuss’s monkey *A. preussi* is shown to be restricted to the southern part of Ebo (Cronin et al. 2018), while our results show that the species is also spread northward into Ndokbou, where its encounter rate is even higher (though not significantly) than that in Ebo. As components of the same Key Biodiversity Area and the same landscape, we recommend that Ebo, Ndokbou, and Makombe should all be considered as a single conservation unit given that they have the same importance for primates, the same hunting and logging threats, and near-identical local stakeholders. Conservation strategies should therefore be designed to protect wildlife and tackle anthropogenic threats across the entire landscape simultaneously.

### Polyspecific primate associations in YKBA

Over 55% of all encounter events with primates identified monospecific groups, very similar to the 56% reported by Astaras et al. (2011) in Korup National Park and the 57% reported by Waltert et al. (2002) in the support near the same area. Semi-terrestrial primate species, including the Preuss’s monkey *A. preussi* and the drill *M. leucophaeus*, occurred mostly in monospecific groups. Unlike Astaras et al. (2011), which found a statistically significant association between the drill *M. leucophaeus* and the red-capped mangabey *Cercocebus torquatus* in Korup National Park, we found no evidence of such association in YKBA. This result may be linked to the very few direct sightings of the two species in our area (11 and 13 sightings for the red-capped mangabey *Cercocebus torquatus* and the drill *M. leucophaeus*, respectively). Similar to their results, the red-capped mangabey *Cercocebus torquatus* in YKBA was mostly found in groups with at least three other primate species, likely an antipredation strategy (Astaras et al. 2011). Other arboreal species were also commonly found in polyspecific groups. The putty-nosed monkey *C. nictitans* formed most of its polyspecific associations with the crowned monkey *C. pogonias* and the red-eared monkey *C. erythrotis*. It also occurred considerably in monospecific groups (over 30% of the encounter events). The species also appeared to occupy the higher canopy layers more frequently than any other species, which may be one of the reasons for its high resilience to hunting. Conversely, the Preuss’s monkey *A. preussi* and the drill *M. leucophaeus* were mostly observed within the forest understory (Astaras et al. 2011; Gonzalez Kirchner 2004), likely making them more vulnerable to gun hunting and snaring (Dempsey et al. 2024). In line with findings from Gonzalez Kirchner (2004), the Preuss’s monkey was mostly found in monospecific groups. We found no considerable difference in the percentage of polyspecific groups of different size across the three areas, corroborating the results from Waltert et al. (2002) who reported no significant difference in the proportion of mixed primate groups between logged and unlogged sites near Korup National Park. Nevertheless, we only recorded mixed groups of five species in Ebo, the conserved site which was unlogged at the time of the surveys. Interestingly, primate group sizes were very similar across the three sites, suggesting compensatory increase in the abundance of some species as the abundance of other species decreases, as suggested by Waltert et al. (2002) and Linder and Oates (2011). We recommend further studies in this landscape to understand primate association patterns and assess how association patterns are influenced by hunting and other human pressures.

## Conclusion

We carried out the most comprehensive primate surveys to date across the YKBA. Our intensive surveys over a whole year demonstrated that the YKBA remains a stronghold for primate conservation. However, our failure to record the Preuss’s red colobus *Piliocolobus preussi* shows that hunting may be driving the most vulnerable primate species to local extinction in the landscape. Our results also showed that encounter rates and polyspecific assemblages of most primate species in this unprotected landscape are very similar to reports from the Korup National Park, a formally protected area in the same ecoregion. This strengthens the importance of the YKBA as a critical area for primate conservation in the Gulf of Guinea biodiversity hotspot. Furthermore, we found that the encounter rates of many primate species were relatively similar between Ebo, Ndokbou, and Makombe, despite historic conservation attention that has mainly focused on Ebo. Ndokbou, and Makombe have been highly overlooked, including in the action plans of many of their primate species. The entire YKBA is currently facing increasing hunting and logging pressures, which are likely to imperil the rich primate community and biodiversity in the landscape. We recommend that conservation actions should be implemented at the level of the entire YKBA to ensure the survival of its primate community and the integrity of their habitat.

## Electronic supplementary material

Below is the link to the electronic supplementary material.

Supplementary material 1 (PDF 297 kb)

## Data Availability

The dataset used for the current study is available from the corresponding author on reasonable request.
