# Supplementary material for: An overlooked hotspot: persistence of large polyspecific assemblages of threatened primates in the unprotected Yabassi Key Biodiversity Area
Source: Primates. 2025 Sep 2;66(6):531–43. doi: 10.1007/s10329-025-01212-5 (PMC12680780; doi:10.1007/s10329-025-01212-5)
Supplement: Supplementary file 1 — Supplementary material 1 (PDF 297 kb) [file 10329_2025_1212_MOESM1_ESM.pdf]

Supplementary material

An overlooked hotspot: persistence of large polyspecific assemblages of threatened primates in the unprotected Yabassi Key Biodiversity Area

Vianny Rodel Vouffo Nguimdo, Ekwoge Enang Abwe, Nelson Ekole Betobe, Bethan Jane Morgan & Matthias Waltert

Corresponding author:

Vianny Rodel Vouffo Nguimdo

Department of Conservation Biology, University of Göttingen, Göttingen, Germany

Cameroon Biodiversity Association, Douala, Cameroon

Vianny Rodel Vouffo Nguimdo; vnguimd@uni-goettingen.de / rodel@eboforest.org

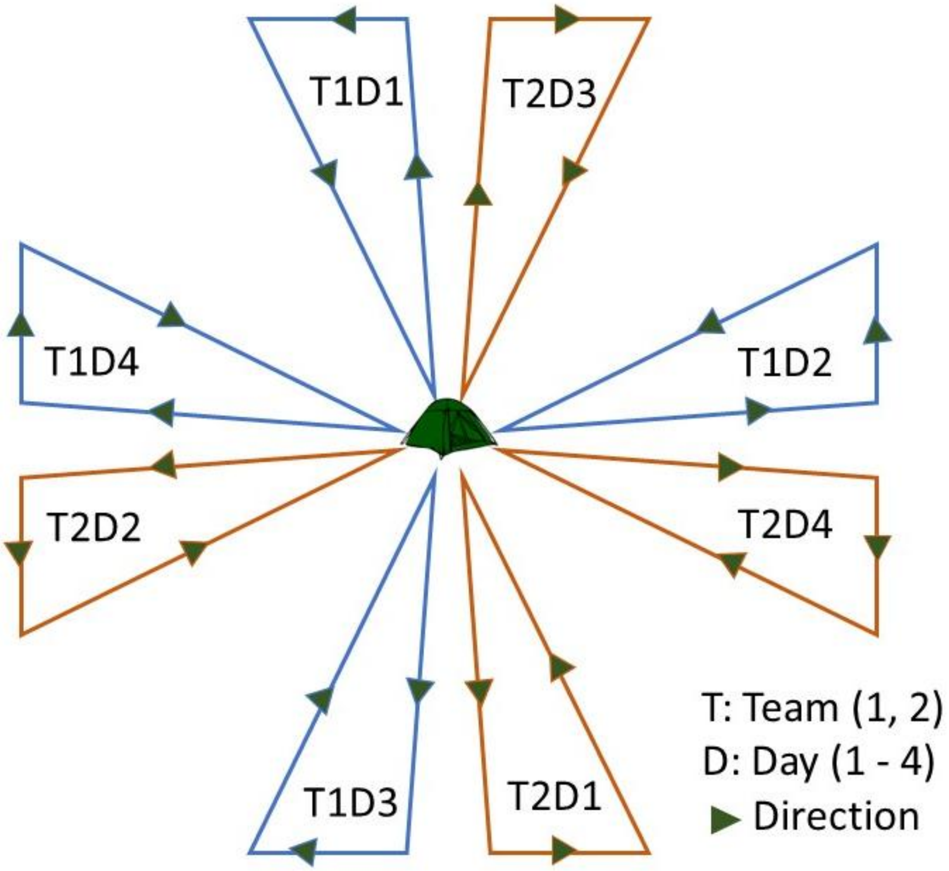

Fig. S1 Illustration of the movement direction of the two survey teams during field activities from the camping site (green tent at the centre). The colours represent each team and the green arrows indicate the movement direction.

Table S1 Results of species association assessment using the package ‘spaa’ in R

Chisq: Chi Square matrix

	A. preussi	*C. erythrotis*	*C. mona*	*C. nictitans*	*C. pogonias*	*C. torquatus*
C. erythrotis	28.721					
C. mona	16.478	3.255				
C. nictitans	50.214	2.434	0.001			
C. pogonias	14.407	15.364	6.188	17.519		
C. torquatus	0.656	1.654	0.126	0.208	3.694	
M. leucophaeus	1.138	1.045	0.457	0.558	1.106	0.009

V: V positive or negative association

	A. preussi	*C. erythrotis*	*C. mona*	*C. nictitans*	*C. pogonias*	*C. torquatus*
C. erythrotis	-0.190					
C. mona	0.151	0.214				
C. nictitans	-0.413	0.095	-0.024			
C. pogonias	-0.016	0.302	0.341	0.238		
C. torquatus	0.452	0.214	0.587	-0.024	0.373	
M. leucophaeus	0.437	0.135	0.540	-0.071	0.278	0.810

Ochiai: Ochiai's index

	A. preussi	*C. erythrotis*	*C. mona*	*C. nictitans*	*C. pogonias*	*C. torquatus*
C. erythrotis	0.077					
C. mona	0.000	0.361				
C. nictitans	0.079	0.517	0.317			
C. pogonias	0.100	0.525	0.370	0.567		
C. torquatus	0.039	0.209	0.132	0.184	0.233	
M. leucophaeus	0.036	0.082	0.040	0.121	0.061	0.000

Jaccard: Jaccard's index

	A. preussi	*C. erythrotis*	*C. mona*	*C. nictitans*	*C. pogonias*	*C. torquatus*
C. erythrotis	0.038					
C. mona	0.000	0.202				
C. nictitans	0.038	0.345	0.162			
C. pogonias	0.052	0.353	0.217	0.381		
C. torquatus	0.014	0.066	0.055	0.051	0.081	
M. leucophaeus	0.014	0.027	0.017	0.036	0.022	0.000

Pearson: Pearson's correlation coefficient

	A. preussi	*C. erythrotis*	*C. mona*	*C. nictitans*	*C. pogonias*	*C. torquatus*	*M. leucophaeus*
A. preussi	1.000	-0.347	-0.268	-0.456	-0.249	-0.074	-0.088
C. erythrotis	-0.347	1.000	0.124	0.106	0.256	0.101	-0.083
C. mona	-0.268	0.124	1.000	0.008	0.168	0.047	-0.066
C. nictitans	-0.456	0.106	0.008	1.000	0.272	0.048	-0.065
C. pogonias	-0.249	0.256	0.168	0.272	1.000	0.142	-0.085
C. torquatus	-0.074	0.101	0.047	0.048	0.142	1.000	-0.050
M. leucophaeus	-0.088	-0.083	-0.066	-0.065	-0.085	-0.050	1.000

AC: Association coefficient

	A. preussi	*C. erythrotis*	*C. mona*	*C. nictitans*	*C. pogonias*	*C. torquatus*
C. erythrotis	-0.753					
C. mona	-1.000	0.072				
C. nictitans	-0.777	0.135	0.017			
C. pogonias	-0.641	0.215	0.243	0.180		
C. torquatus	-0.618	0.026	0.021	0.010	0.044	
M. leucophaeus	-0.677	-0.430	-0.588	-0.266	-0.527	-1.000
